# Oral *Lactobacillus reuteri *LR06 or *Bifidobacterium* BL5b supplement do not produce analgesic effects on neuropathic and inflammatory pain in rats

**DOI:** 10.1002/brb3.1260

**Published:** 2019-03-06

**Authors:** Jiangju Huang, Chuanlei Zhang, Jian Wang, Qulian Guo, Wangyuan Zou

**Affiliations:** ^1^ Department of Anesthesiology Xiangya Hospital, Central South University Changsha China

**Keywords:** *Bifidobacterium*, chronic pain, gut microbiota, *Lactobacillus reuteri*, probiotic

## Abstract

**Background:**

Previous studies have reported that certain bacteria exert visceral antinociceptive activity in visceral pain and may also help to relieve neuropathic and inflammatory pain. Objective: The aim of this study was to explore the analgesic effect of *Lactobacillus reuteri* LR06 (LR06) or *Bifidobacterium *BL5b (BL5b) in chronic pain in vivo.

**Design:**

Rats were randomly assigned into four groups: sham, Chronic Constriction Injury (CCI)/Complete Freund's Adjuvant (CFA) + control, CCI/CFA + LR06, and CCI/CFA + BL5b. Rats from the probiotic groups were treated with 1 x 10^9 ^cfu (LR06 or BL5b) daily through gavage for 14 days after a pain model was successfully established. Mechanical and thermal hyperalgesia were used to assess the analgesic effect of the probiotics. Iba1 was used to verify the microglial inflammatory reaction in the different groups.

**Results:**

The results showed that probiotics *L. reuteri *LR06 or *Bifidobacterium *BL5b had no significant antinociception effects in chronic pain rats. The chronic pain‐induced activation of microglia (Iba1) was not relieved by probiotics in CCI/CFA‐induced neuropathic or inflammatory pain rats.

**Conclusion:**

Our results suggested that *L. reuteri *LR06 or *Bifidobacterium *BL5b had no antinociceptive effects on CCI‐induced neuropathic pain and CFA‐induced inflammatory pain in rats.

## 1 INTRODUCTION

Chronic pain is defined as continuous, long‐term pain lasting more than 12 weeks. Chronic pain is common worldwide, with an estimated rate ranging from 8% to 60% (Phillips, 2009), and profoundly impacts the overall quality of life and mental health without effective management (Breivik, Collett, Ventafridda, Cohen, & Gallacher, 2006). Integrated multidisciplinary management of pain‐based biopsychosocial models has proven to be clinically effective but has not been made widely available (Kress et al., 2015).

Probiotic, live bacteria that confer health benefits to the host when administered in a sufficient amount, have been reported as a new method to improve our daily lifestyle (Sharma & Im, 2018). With the development of research methods to examine the gut microbiome, increasing evidence has demonstrated that the enteric microbiome is associated not only with the pathogenesis of disease but also with the therapeutic potential of probiotics in disease treatment (Yu, Liu, Li, Wen, & He, 2018).

The clinical therapeutic potential of probiotic bacteria, particularly *Lactobacilli reuteri *and *Bifidobacteria*, is the focus of considerable interest in many fields, with chronic pain treatment being no exception (Quigley, 2005). Studies have indicated positive therapeutic effects of probiotics, including in irritable bowel syndrome (IBS) (Ford, Harris, Lacy, Quigley, & Moayyedi, 2018), obesity (Emond, Golding, & Peckham, 1989), autism spectrum disorders (ASDs) (Grimaldi et al., 2018), diabetes mellitus (Balakumar et al., 2018), asthma (Fonseca et al., 2017), and cognitive and emotional impairments in fibromyalgia (Roman et al., 2018). In addition, increasing evidence has shown that the gut microbiota dramatically impacts visceral pain (Luczynski et al., 2017; Perez‐Burgos et al., 2015; SM, Dinan, & Cryan, 2017), functional abdominal pain (FAP) (Jadresin et al., 2017), chronic prostatitis/chronic pelvic pain syndrome (Shoskes et al., 2016), inflammatory pain induced by formaldehyde (Amaral et al., 2008), and chemotherapy‐induced pain (Shen et al., 2017). However, few studies have focused on the relationship between probiotics and neuropathic and inflammatory pain. Therefore, in this study, we hypothesized that oral supplementation with *Lactobacillus reuteri* LR06 or *Bifidobacterium *BL5b can relieve CCI‐induced neuropathic pain and CFA‐induced inflammatory pain in rats.

## 2 MATERIALS AND METHODS

### 2.1 Animals

Adult Sprague‐Dawley (*SD*) rats (weighing 220–250 g, male) were obtained from the Animal Experiment Center of Xiangya School, Medicine of Central South University. All animals were raised under a 12:12 hr light cycle and had ad libitum access to food and water. All experimental operations adhered to the National Institute of Health Guide on the Care and Use of Laboratory Animals. All protocols were approved by the Animal Care Committee of Xiangya Hospital, Central South University and were in accordance with the guidelines provided by the National Institute of Health (Zimmermann, 1983).

### 2.2 Pain models

The CCI model was established based on a previous description (Bennett & Xie, 1988). Briefly, rats were anesthetized with 3% pentobarbital sodium (30 mg/kg; i.p.). To prevent blood circulation interruption, four chromic gut ligatures were loosely tied (4.0 silk) around the nerve at 1 mm intervals. The sham group underwent the same operation but was not ligated. After nerve surgery, the muscle and skin were closed separately.

To induce inflammatory pain, rats received 100 µl subcutaneous (s.c.) injection of complete Freund's adjuvant (Sigma, USA) into the left hind paw. Saline was used for the vehicle sham groups (Roca‐Lapirot et al., 2018). The animals were randomly assigned to experimental groups, untreated or probiotically treated, and submitted to behavioral tests every two days, and body weight (g) was measured before every behavioral test.

### 2.3 Probiotics

LR06 and BL5b were purchased from New Biochemistry Technology (Jiangsu, China) and suspended in drinking water at a density of 1.0 x 10^9 ^cfu/ml. Rats were gavaged with 1 ml saline, LR06 or BL5b at 9:00 a.m. once a day.

### 2.4 Mechanical and thermal hyperalgesia

All behavioral tests were performed in a blinded manner. Behavioral testing was conducted prior to surgery and on days 3, 5, 7, and 14 following surgery. Animals were tested in clear plastic cages (22 × 12 × 12 cm) with a wire mesh bottom and habituated for a few minutes. Mechanical thresholds were assessed by stimulation with von Frey filaments (ranging from 0.14 to 15 g) (Stoelting, USA) applied to the left hind paw plantar surface as previously reported (Zou et al., 2017). The mechanical threshold of each animal was the average of three measurements.

To test the thermal withdrawal latency, rats were placed in a plantar test instrument (Ugo Basile, Italy), which was a transparent, square and bottomless acrylic box, and allowed to adapt for 30 min. Three heat stimuli were applied, and the response was recorded. Stimulation of each hind paw was repeated at 5 min intervals. A cutoff time was set at 30 s to prevent tissue damage. The results obtained for each rat were expressed in seconds as the mean of three thermal withdrawal latencies.

### 2.5 Immunofluorescence

After perfusion with PBS and 4% paraformaldehyde when pain models were established for 14 days, the spinal cord was fixed, dehydrated, and embedded in OTC (Leica, USA). Coronal sections 10 µm thick were cut on a microtome (Leica). The sections were rinsed with PBS and incubated with 5% normal goat serum diluted in PBS for 1 hr at room temperature. Then, the cells were incubated with rabbit anti‐Iba1 (1:400, Wako, Japan) antibody diluted in PBS overnight at 4°C. The next day, the cells were incubated with Alexa Fluor 488‐conjugated secondary antibody (1:400, Jackson, USA) for 2 hr at room temperature and observed on a Leica observer microscope connected to a computer (Leica).

### 2.6 Statistical analysis

The data are presented as the mean ± standard deviation. Behavioral results and staining data were examined using two‐way repeated‐measures ANOVA followed by Bonferroni correction testing. Statistical analyses used the GraphPad Prism software v7.0. The significance level of the test results was set at *p* < 0.05.

## 3 RESULTS

### 3.1 Effects of *L. reuteri* LR06 or *Bifidobacterium *BL5b on body weight in CCI‐ and CFA‐treated rats

**Figure 1:**
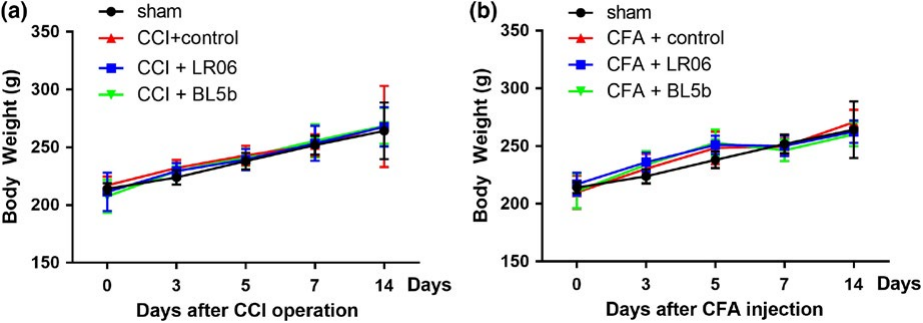
*Lactobacillus reuteri *LR06 or *Bifidobacterium *BL5b have no significant impacts on rat body weight. (a) Body weight of rats from each CCI group and (b) CFA group as indicated. Data are indicated as the mean ± *SD*. *n* = 6 in each group. Two‐way repeated‐measures ANOVA followed by Bonferroni correction testing

Body weight was monitored before every behavioral test. In this study, the rats in the sham group exhibited an increase in weight during the experiment. However, the body weights in the CCI groups (Figure 1a) and CFA groups (Figure 1b) treated with LR06 or BL5b were not significantly different from those in the control group (*p* < 0.05).

### 3.2 Effect of *L. reuteri *LR06 OR* Bifidobacterium *BL5B on mechanical and thermal hyperalgesia in CCI‐treated rats

**Figure 2:**
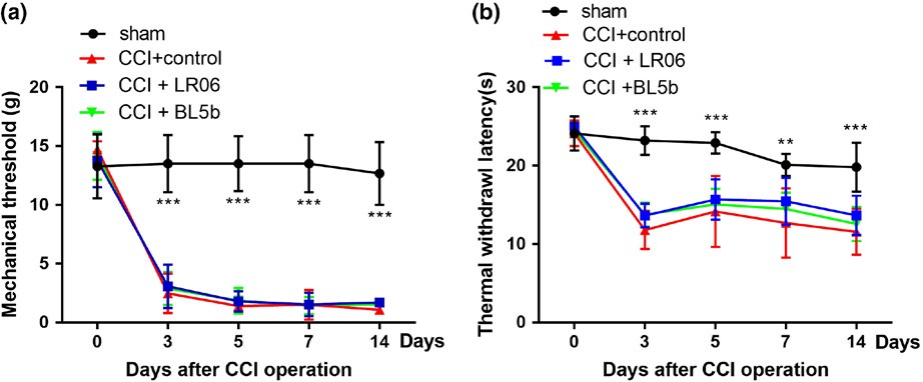
Gavage‐fed *Lactobacillus reuteri *LR06 or *Bifidobacterium* BL5b show no significant pain relief in CCI‐treated rats. (a) The effects of LR06 and BL5b on thermal hyperalgesia were evaluated by von Frey filaments. (b) The effect of LR06 and BL5b on thermal hyperalgesia was evaluated by thermal withdrawal latency. *n* = 6 for each group. Error bars represent the mean ± *SD*, **p < 0.01 and ***p < 0.001 compared with the sham group. Two‐way repeated‐measures ANOVA followed by Bonferroni correction testing

Rats that underwent CCI surgery developed pain sensitization. As shown in Figure 2, no significant differences were observed in mechanical threshold or thermal withdrawal latency between groups before CCI (*p* > 0.05). The mechanical threshold and thermal withdrawal latency were lower in the CCI group than in the sham beginning at day 3 post operation (*p* < 0.001), which demonstrated the successful establishment of the CCI model. This decrease persisted until day 14 post surgery (*p* < 0.001), which is consistent with previous studies (Lim, Wang, Zhang, Tian, & Mao, 2009).

To test the effect of* L. reuteri* LR06 and *Bifidobacterium *BL5b on neuropathic pain behavior, rats were assigned to four groups after the CCI model was established: sham, CCI + control, CCI + LR06*,* and CCI + BL5b. Rats were given 1 × 10^9 ^cfu probiotics or the same volume of vehicle each morning for 14 days by gavage. The mechanical threshold (Figure 2a) and thermal withdrawal latency (Figure 2b) were examined to test the analgesic effect of the two probiotics on chronic pain. The data showed that there were no significant differences between the CCI groups (*p* > 0.05), which demonstrated that the probiotics we chose had no significant influence on CCI‐induced neuropathic pain.

### 3.3 Effects of *L. reuteri *LR06 OR* Bifidobacterium *BL5B on mechanical and thermal hyperalgesia in CFA

**Figure 3:**
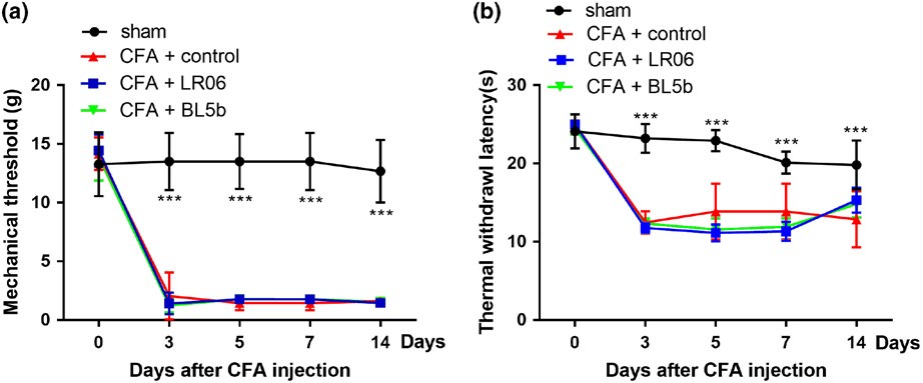
No antinociceptive effects of *Lactobacillus reuteri *LR06 or *Bifidobacterium *BL5b on CFA‐induced mechanical allodynia or thermal hyperalgesia. The effect of LR06 and BL5b on CFA‐induced mechanical allodynia (a) and hyperalgesia (b) were evaluated. The data are expressed as the mean ± *SD* and ***p < 0.001 indicate significant differences from the sham group. Two‐way repeated‐measures ANOVA followed by Bonferroni correction testing

The mechanical threshold of the CFA groups was significantly decreased after CFA injection, and this effect lasted for at least 14 days (*p* < 0.001). The CFA rats exhibited a significant decrease in thermal withdrawal latency over the same time frame (*p* < 0.001). These data showed that the rats developed mechanical allodynia and thermal hyperalgesia after CFA injection (Figure 3).

To evaluate the role of LR06 or BL5b on the CFA‐induced nociceptive response, 1 × 10^9 ^cfu LR06 or BL5b were administered by gavage each morning for 14 days from 1 day to 14 days after CFA injection. The rats in the control group were given the same volume of drinking water. Mechanical allodynia (Figure 3a) and thermal hyperalgesia (Figure 3b) in the CFA + LR06 and CFA + BL5b groups were significantly greater than those in the sham group (*p < *0.001), but no significant differences were observed compared to the CFA + control group (*p* > 0.05).

### 3.4 Effect of *L. reuteri *LR06 and *Bifidobacterium *BL5B on IBa1 protein expression in the rat spinal cord

**Figure 4:**
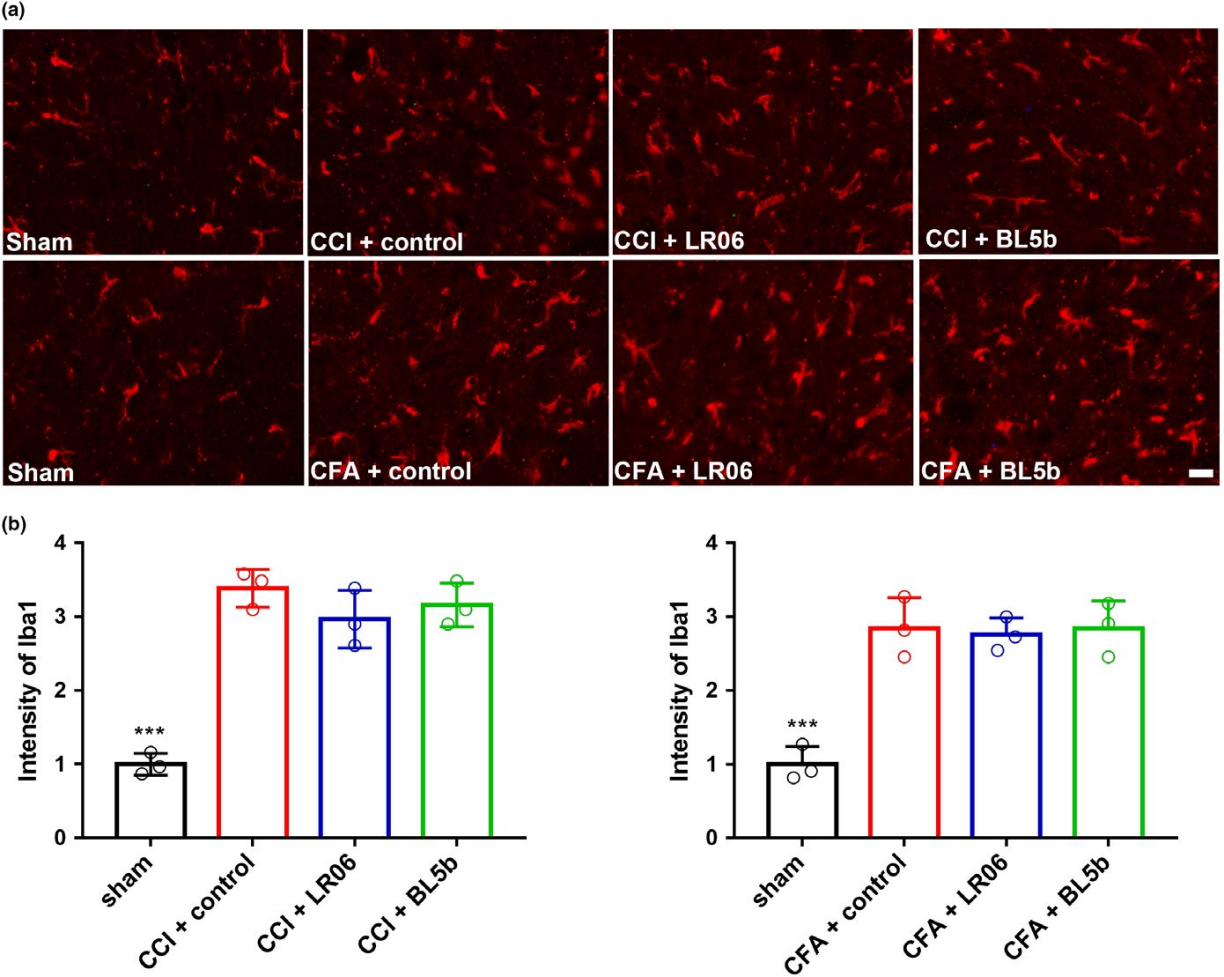
Effects of *Lactobacillus reuteri* LR06 or *Bifidobacterium *BL5b on the expression levels of Iba1 following chronic pain in the spinal cord of rats. (a) Microglia marker Iba1 in the spinal cord in the indicated groups was detected by immunofluorescence on day 14 and was visualized by fluorescence microscopy. Representative images are shown. (b) The immunofluorescence staining of Iba1 in CCI or CFA groups was significantly increased compared to that in each sham group, but there were no significant differences between the different CCI/CFA groups. ***p < 0.001 indicates significant differences from the sham group. Two‐way repeated‐measures ANOVA followed by Bonferroni correction testing. Scale bar = 20 μm (b)

Previous research found that antibiotic‐induced gut dysbiosis exacerbated lesion pathology and intraspinal inflammation, and the magnitude of the microglia reaction was greater in spinal cord injury (SCI) mice (Kigerl et al., 2016). Microglial activation, indicated by expression of the marker Iba1 (ionized calcium‐binding adapter molecule 1), in the spinal cord is necessary for the development of nociceptive hypersensitivity after neuropathic pain and inflammation pain (Grace et al., 2016). The spinal cord receives sensory information from primary afferent Aб and C fibers after nociceptive stimuli, and this innervation is concentrated in the superficial dorsal horn, an important area in chronic pain research. We examined the L4‐L5 spinal cord sections of experimental and control rats for inflammatory reactions by assessing the expression of Iba1. Experimental rats had a significantly higher expression of Iba1 than the sham group, but there were no differences among the CFA and CCI rats given vehicle or probiotics (Figure 4).

## 4 DISCUSSION

Our data showed that the *L. reuteri *LR06 and *Bifidobacterium *BL5b have no effective pain relief in CCI‐induced neuropathic pain or CFA‐induced inflammatory pain.

Chronic pain has been classified into three principle categories: nociceptive pain, neuropathic pain, and mixed pain (Baron, Binder, & Wasner, 2010). Recently, increasing research has suggested that glutamine (Liu, Guo, Huang, Zou, & Song, 2013) and BDNF (brain‐derived neurotrophic factor) (Zhu et al., 2014) bind to postsynaptic AMPA/NMDA ionotropic receptors, resulting in increased intracellular Ca^2+^, which triggers protein kinase C (PKC) (Zou et al., 2012) and calmodulin‐dependent kinase Ⅱ (CaMKⅡ)‐mediated signaling cascades and activation of the TLR4 (toll‐like receptor 4) pathway (Kuang et al., 2012), which contribute to mechanical and thermal hyperalgesia. However, no research about the effects of probiotics on neuropathic pain and inflammatory pain have been reported. Therefore, the identification of a novel strategy to alleviate these types of pain is still imperative.

The gut microbiome comprises more than 1,000 species and 7,000 strains dominated mainly by bacteria, viruses and yeast (Human Microbiome Project, 2012). Humans have a symbiotic relationship with this microbial community (Virili, Fallahi, Antonelli, Benvenga, & Centanni, 2018). The gut microbiome cluster diversity in patients with pelvic pain syndrome has been found to be significantly lower than that in controls (Shoskes et al., 2016) and is a primary actor in Crohn's disease pathogenesis (Brusaferro et al., 2018). Some research has reported that the gut microbiota impacts normal visceral pain perception (Pusceddu & Gareau, 2018). However, there is little research concerning the gut microbiota and neuropathic and inflammatory pain.

In both animal and human trials, probiotics have been implemented for the prevention and treatment of a wide variety of systemic conditions, such as rheumatoid arthritis (de Oliveira, Leite, Higuchi, Gonzaga, & Mariano, 2017) and systemic lupus erythematosus (SLE) (Esmaeili et al., 2017). The mechanism of the effects of probiotic treatment may include exclusion of pathogenic microorganisms and immune system modulation, such as suppression of anti‐inflammatory cytokines IL‐10 or TGF‐β (Lavasani et al., 2010) and pro‐inflammatory cytokines (Kwon et al., 2013). In addition, in the dorsal horn of the spinal cord, activated glial cells, such as microglia, can affect pain transmission by releasing extracellular signaling molecules after peripheral nerve injury and have been identified as one of the key players in the pathogenesis of neuropathic pain (Isami et al., 2018). Therefore, in the study, we chose the microglia marker Iba1 to investigate the effect of *L. reuteri *LR06 and *Bifidobacterium *BL5b on the inflammatory reaction in CFA rats.

The *Lactobacillus casei* and *Bifidobacteria *groups are some of the most widely researched and applied probiotic species (Hill et al., 2018). The health‐promoting capabilities of these probiotics have been documented in child asthma (Kalliomaki et al., 2001), mania (Dickerson et al., 2018), and obesity (Luoto, Kalliomaki, Laitinen, & Isolauri, 2010). Previous research has shown that administration of *Lactobacillus acidophilus* in the gut results in analgesic effects in rodents similar to those observed with morphine (Rousseaux et al., 2007) and that the *Escherichia coli* strain Nissle 1917 provided analgesia for the visceral pain associated with irritable bowel syndrome (Perez‐Berezo et al., 2017). Furthermore, *Lactobacillus casei* Shirota relieves pain after single rib fracture (Lei et al., 2018). *L. reuteri *has a positive effect on the incidence of diarrhea with no effect on respiratory illness in children (Weizman, Asli, & Alsheikh, 2005) and inhibits the onset of colitis in transgenic IL‐10 deficient mice (Madsen, Doyle, Jewell, Tavernini, & Fedorak, 1999). Importantly, live *L. reuteri and Bifidobacteria *can easily be obtained from yogurt or other dairy products.

Our data showed that *L. reuteri* LR06 or *Bifidobacteria* BL5b have no analgesic effect on CCI‐induced neuropathic pain and CFA‐induced inflammatory pain. Some reasons for this observation are as follows: first, the probiotics we chose may not have antinociceptive effects. A study reported that the efficacy of prebiotics should be assessed in subgroups using a specific type of prebiotic (McFarland & Goh, 2018). Second, the gavage method used here to administer the probiotics could not ensure administration of an adequate number of living microorganisms, which, upon ingestion in adequate numbers, act in the stomach acid. The largest trial examining *Bifidobacterium infantis *35,624 was a dose‐ranging study and demonstrated the efficacy of a capsule at a dose of 1 × 10^8 ^cfu, that was stable and amenable to widespread use (Bjerg et al., 2014; Whorwell et al., 2006). Third, most of the early research on the microbiome was conducted in germ‐free mice, which are born in sterile conditions and free of all microorganisms. Because these mice can be selectively inoculated with microbes of interest, experiments with germ‐free mice have yielded intriguing clues about the possible influence of the gut microbiome on behavior and neurodevelopment (Konkel, 2018). Last but not least, the methods used to measure allodynia may not have been sufficiently sensitive to measure the antinociceptive function of the probiotic.

Autologous fecal microbiome transplantation (aFMT) can induce a rapid and near‐complete recovery within days of administration (Suez et al., 2018). Therefore, aFMT may be a new way to ameliorate chronic pain. Prebiotic dietary fibers increase the bulking capacity, improve transit times, and even stimulate the growth and activity of resident beneficial bacteria (Vermeulen et al., 2018).

Although *L. reuteri *LR06 and *Bifidobacterium *BL5b did not have significant effects on neuropathic pain and inflammatory pain in rats, the microbiome is ubiquitous across species and variable among individuals and is a promising avenue of research for investigating chronic pain mechanisms in the context of individual differences (Davidson, Cooke, Johnson, & Quinn, 2018). In conclusion, our results suggested that neither *L. reuteri *LR06 nor *Bifidobacterium *BL5b had beneficial antinociceptive effects on CCI‐induced neuropathic pain and CFA‐induced inflammatory pain in rats.

## CONFLICT OF INTEREST

The authors declare that they have no competing interests.

## AUTHORS’ CONTRIBUTIONS

JH performed the experiments, collected and analyzed the data, and drafted the manuscript. CZ and WJ performed the experiments and analyzed the data. WZ designed the study and analyzed the data. WZ and QG revised the manuscript. All authors read and approved the final manuscript.
